# Drug–drug interactions in the management of non-tuberculous mycobacterial infections

**DOI:** 10.3389/fmicb.2024.1468383

**Published:** 2024-09-05

**Authors:** Kazuaki Takeda, Takahiro Takazono, Hiroshi Mukae

**Affiliations:** ^1^Department of Respiratory Medicine, Nagasaki University Hospital, Nagasaki, Japan; ^2^Department of Infectious Diseases, Nagasaki University Graduate School of Biomedical Sciences, Nagasaki, Japan; ^3^Department of Respiratory Medicine, Nagasaki University Graduate School of Biomedical Sciences, Nagasaki, Japan

**Keywords:** non-tuberculous mycobacterial infection, drug interaction, chronic pulmonary aspergillosis, antiretroviral therapy, adverse event

## Abstract

Non-tuberculous mycobacterial pulmonary disease (NTM-PD) is a refractory chronic respiratory infectious disease and its prevalence is increasing globally. The standard treatment regimen for NTM-PD involves long-term multidrug therapy including macrolides. The incidence of adverse events is high given the advanced age of many NTM-PD patients. In addition, drug–drug interactions under coexisting conditions add additional complexity. Despite guidelines advocating multidrug therapy for NTM-PD, low adherence rates probably owing to the relatively frequent adverse events and drug interactions. An appropriate treatment regimen can improve the bacteriological response rates, reduce the development of macrolide resistance, and mitigate adverse events. Of particular concern are the interactions arising from new complications that develop with NTM-PD. Notably, chronic pulmonary aspergillosis occasionally co-infects NTM-PD, which can lead to poor prognosis. The primary therapeutic modality for chronic pulmonary aspergillosis is the azoles. However, the interaction with rifamycin is problematic, making it challenging to continue standard treatment for NTM-PD and requiring drug adjustments. The implications of rifamycin extend beyond chronic pulmonary aspergillosis, impacting various other diseases such as those requiring immunosuppressive agents and AIDS patients requiring antiretroviral therapy. Hence, a comprehensive consideration of drug interactions is imperative for the initiation of NTM-PD treatment. This mini-review focuses on drug–drug interactions in a multidrug regimen for NTM-PD and discusses the essential points to be considered in the treatment of NTM.

## 1 Introduction

Non-tuberculous mycobacterial pulmonary disease (NTM-PD) is a refractory chronic respiratory infectious disease in patients with pulmonary structural abnormalities such as bronchiectasis, cystic pulmonary fibrosis, and chronic obstructive pulmonary disease (COPD), as well as in those with mild immunosuppression caused by the use of oral steroids, immunosuppressants, and biological agents. The incidence of NTM-PD is rising, and the mortality rates associated with the disease are increasing globally (Adjemian et al., 2012; Namkoong et al., 2016; Harada et al., 2021; Dahl et al., 2022). Long-term multidrug therapy, which includes macrolides, is the standard treatment for NTM-PD, but this treatment can sometimes cause problematic drug–drug interactions. The treatment duration for NTM-PD is longer than that for pulmonary tuberculosis, and the frequency of adverse events is higher because many of the treated patients are elderly. In Japan, the continuation rates of standard treatment at six and 12 months are 59 and 41%, respectively (Morimoto et al., 2019). Although guidelines recommend multidrug therapy, low compliance rates have been reported, possibly because of the high frequency of adverse events (Adjemian et al., 2014; van Ingen et al., 2017). Patients with macrolide-resistant *Mycobacterium avium intracellulare* (MAI) PD have poor prognosis; therefore, it is crucial to select a treatment regimen that does not induce macrolide resistance (Kobashi et al., 2006; Griffith et al., 2006). This mini-review provides an overview of drug–drug interactions that can occur in NTM-PD multidrug therapy.

## 2 The drug-drug interactions in NTM-PD treatment

The drug–drug interactions between the NTM-PD drugs are presented in Table 1. Based on the results of a randomized controlled trial (RCT) that showed the efficacy of multidrug therapy, including clarithromycin (CAM), in treating AIDS-MAI bacteremia, CAM has become the standard treatment for MAI disease (Shafran et al., 1996). Subsequently, RCT were conducted to evaluate the effects of CAM and clofazimine (CFZ) with and without ethambutol (EB) on AIDS-MAI bacteremia. The results showed that regimens that included EB significantly reduced the relapse rate and emergence of CAM-resistant strains (Dube et al., 1997). In another RCT, the addition of rifabutin (RBT) to CAM and EB did not improve the bacteriological response rate in AIDS patients with disseminated MAI infection, but improved survival and inhibited the development of macrolide resistance (2% RBT, 14% placebo; Gordin et al., 1999). These findings indicate that both EB and RBT can inhibit macrolide resistance in AIDS patients. However, a prospective trial comparing CAM and EB with and without CFZ resulted in significantly higher mortality in the CFZ group (61% CFZ, 38% placebo; Chaisson et al., 1997). Nevertheless, a recent retrospective study of non-AIDS MAI-PD patients showed a higher rate of culture conversion in the CFZ group than in the rifampicin (RFP) group (100% vs. 71%), which differs significantly from the results of AIDS-MAI bacteremia (Jarand et al., 2016).

**Table 1:** Drug–drug interactions with non-tuberculous mycobacterial infection treatment.

Antimicrobial agents	Disease / Mycobacterium	Concomitant drugs	Interactions	Study design	References
Standard regimen	HIV MAI bacteremia	CAM, EB, RBT	CAM-containing regimen improves survival curve compared with regimen without CAM.	prospective study	Shafran et al. (1996)
	HIV MAI bacteremia	CAM, CFZ ± EB	EB-containing regimen reduces relapses and emergence of CAM resistance.	prospective study	Dube et al. (1997)
	HIV MAI bacteremia	CAM, EB ± CFZ	CFZ-containing regimen worsens mortality.	prospective study	Chaisson et al. (1997)
	non-HIV MAI-PD	macrolide, RFP ± EB	EB-containing regimens reduce macrolide resistance compared to macrolide monotherapy.	retrospective study	Griffith et al. (2006)
	non-HIV MAI-PD	CAM, RFP, EB ± SM	SM-containing regimen improves the sputum conversion rate.	prospective study	Kobashi et al. (2007)
	non-HIV MAI-PD		No significant difference in microbial outcomes between CAM and AZM regimens.	meta-analyses	Pasipanodya et al. (2017)
	NB MAI-PD	macrolide, EB, RFP	Intermittent therapy reduced EB discontinuation caused by adverse events.	prospective study	Jeong et al. (2015)
	refractory MAI-PD	GBT ± ALIS	ALIS improves culture conversion for refractory MAI-PD.	prospective study	Griffith et al. (2018)
EB	disseminated MAI	CAM	Combined EB plus CAM regimen does not increase CAM-resistant MAI.	*in vivo*	Bermudez et al. (1996)
	MAI	CAM, RFP, CPFX	The combinations of EB with CAM, RFP, and CPFX show synergistic activities against 48, 43, and 71% of the strains, respectively.	*in vitro*	Kent et al. (1992)
	Macrolide-resistant MAI	CAM	64% of macrolide-resistant MAI patients are treated with inappropriate regimens without EB.	retrospective study	Morimoto et al. (2016)
RFP	MAI-PD	CAM, AZM	RFP decreases Cmax of CAM 68%. RFP decreases Cmax of AZM 23%.	retrospective study	van Ingen et al. (2012a)
	non-HIV MAI-PD	CAM, EB	The regimen without RFP does not increase the macrolide resistant and decreases adverse events.	prospective study	Miwa et al. (2014)
RBT	MAI-PD	CAM, AZM	RBT increases Cmax of CAM 7%. RBT increases Cmax of AZM 49%.	retrospective study	van Ingen et al. (2012a)
	HIV disseminated MAI	CAM, EB	RBT decreases the development of CAM-resistance.	prospective study	Gordin et al. (1999)
	HIV MAI	CAM	RBT decreases AUC of CAM 44%. CAM increases AUC of RBT 99%.	prospective study	Hafner et al. (1998)
	HIV MAI prophylaxis	CAM	CAM increases the rate of uveitis development.	prospective study	Benson et al. (2000)
	*M. abscessus*	CAM, AZM, TGC	The combinations of RBT with CAM, AZM and TGC shows synergistic activity.	*in vitro*	Pryjma et al. (2018)
	*M. abscessus*	CAM	The combination of CAM and RBT is synergistic and suppresses of *erm41* expression.	*in vitro*	Aziz et al. (2020)
CFZ	MAI-PD	CAM, EB	CFZ-containing regimen significantly improves culture conversion rate compared with RFP regimen (100% vs. 71%).	retrospective study	Jarand et al. (2016)
	MAI	AMK	The addition of CFZ with AMK improves bacterial outcomes *in vivo.*	*in vivo*	Gangadharam et al. (1988)
	MAI, *M. abscessus*	AMK	The combination of CFZ and AMK shows synergistic activities against *M. abscessus* and MAI.	*in vitro*	van Ingen et al. (2012b)
	MAI, *M. abscessus*	CAM, AMK	The combinations of CFZ with CAM and AMK show synergistic activities against *M. abscessus* and MAI.	*in vitro*	Ferro et al. (2016)
OMD	*M. abscessus*	CAM	The combination of OMD and CAM showes synergistic activity. The combination of OMD and CAM improves bacterial outcomes *in vivo.*	*in vitro*; *in vivo*	Bich Hanh et al. (2021)
	*M. abscessus*	CAM, RBT	The combinations of OMD with CAM and RBT show synergistic activities against 75.8 and 76.9% of the strains, respectively.	*in vitro*	Fujiwara et al. (2023)

ALIS, amikacin liposome inhalation suspension; AMK, amikacin; AUC, area under the curve; AZM, azithromycin; CAM, clarithromycin; CFZ, clofazimine; Cmax, maximum plasma conentration; CPFX, ciprofloxacin; EB, ethambutol; GBT, guideline-based therapy; MAI, Mycobacterium avium intracellulare; MAI-PD, Mycobacterium avium intracellulare-pulmonary disease; OMD, omadacycline; RBT, rifabutin; RFP, rifampicin; SM, streptomycin: TGC, tigecycline.

Several studies have been conducted on MAI-PD in non-AIDS patients, following the results of a prospective RCT on AIDS-MAI bacteremia. A retrospective study of macrolide-resistant MAI-PD found macrolide monotherapy and macrolide and quinolone dual therapy to be risk factors for macrolide resistance (Griffith et al., 2006). Furthermore, macrolide resistance is inhibited in patients treated with macrolides, EB, and rifamycin. In another retrospective study of macrolide-resistant MAI disease, 64.4% of the patients were treated with a regimen that did not include EB prior to therapy (Morimoto et al., 2016). The addition of EB to CAM has been shown to suppress CAM resistance in MAI *in vitro* and *in vivo* (Kent et al., 1992; Bermudez et al., 1996). These results suggest that EB plays a crucial role in the suppression of the emergence of macrolide resistance. This may be due to the fact that EB has additive and synergistic effects with many of the antimicrobial agents (Kent et al., 1992). Since macrolide monotherapy easily induces macrolide resistance, guidelines in the United States, United Kingdom, and Japan recommend macrolide as the key drug and a combination of EB and RFP as the standard regimen (Daley et al., 2020; Haworth et al., 2017). In a randomized, double-blind, comparative study of three-drug therapy plus streptomycin (SM), the culture conversion rate in the SM group was 71.2%, which was higher than that in the placebo group (50.7%; Kobashi et al., 2007). Based on these results, guidelines recommend the use of concomitant aminoglycosides such as SM in severe cases (Daley et al., 2020). In an RCT that included amikacin liposome inhalation suspension (ALIS) in guideline-based therapy (GBT) for refractory MAI-PD, ALIS significantly improved the culture conversion rate after 6 months of treatment (Griffith et al., 2018).

Many drugs commonly used in standard therapies exhibit drug–drug interactions. For instance, RFP and RBT are potent CYP3A4 inducers that significantly decrease blood CAM concentrations (van Ingen et al., 2012a; Miwa et al., 2014). Azithromycin (AZM) is less affected by RFP than CAM, exhibiting a 23% reduction in Cmax (van Ingen et al., 2012a). Although RBT also induces CYP3A4-inducing effects, its impact is less than that of RFP, with a limited reduction in CAM and AZM concentrations (van Ingen et al., 2012a; Hafner et al., 1998). In contrast, CAM significantly increased RBT concentrations, and the concomitant use of CAM considerably increased the incidence of uveitis (Hafner et al., 1998; Benson et al., 2000). In a retrospective study comparing CAM and EB with and without RFP, the rate of treatment interruption due to adverse events was significantly lower in the group without RFP (Miwa et al., 2014). Conversely, the culture conversion rate was higher in the RFP-free group, although the difference was not statistically significant, and the macrolide resistance was not substantially different between the two groups. RFP has not demonstrated efficacy against MAI *in vitro* or *in vivo*, raising questions regarding its significance in NTM treatment (van Ingen et al., 2024). Currently, a prospective study (NCT 03672630) is underway in the United States to compare the efficacy of AZM and EB with and without RFP, which may clarify the significance of adding rifamycins to standard therapy. Additionally, an ongoing RCT compared RFP and CFZ as concomitant drugs with AZM and EB; however, the results await (Zweijpfenning et al., 2024). A global clinical trial (NCT04630145) using bedaquiline (BDQ) as a concomitant drug has recently been initiated. Although BDQ has no significant drug interactions, potential concerns regarding QT prolongation may arise from its interaction with macrolides. Therefore, it is crucial to closely monitor the results of safety evaluation trials.

Preventing the interruption of EB caused by adverse events helps to prevent macrolide resistance in MAI. In a retrospective study comparing daily and intermittent dosing for non-cavitary MAI-PD, there was a significant decrease in EB interruption owing to adverse events in the intermittent group (24% vs. 1%; Jeong et al., 2015). However, there was no significant difference in the culture conversion rate between the two groups (76% vs. 67%), and the rate of macrolide resistance was lower in the intermittent group. Another retrospective study comparing EB ≥ 12.5 mg/kg/day to <12.5 mg/kg/day showed a significant reduction in the incidence of EB-induced ocular neuropathy at lower dose group (20% vs. 3%), with similar rates of macrolide resistance and culture conversion (Ando et al., 2021). Lower-dose EB administration is also a treatment option, although the extent to which reducing EB will help prevent macrolide resistance remains unclear. The exact mechanism how EB suppresses the emergence of macrolide resistance remains unclear; however, it is thought that EB exerts its effects on mycobacterial cell walls, thereby increasing the penetrability of combination agents (Källenius et al., 1989; Hoffner et al., 1989).

Quinolones are recommended in the guidelines for RFP-resistant *M. kansasii* and *M. xenopi* but not for MAI-PD (Daley et al., 2020). A retrospective study was conducted in which moxifloxacin (MFLX) was added to patients with MAI-PD who failed to achieve negative conversion after 6 months of standard therapy. In this study, 29% of the patients who received additional MFLX achieved culture conversion (Koh et al., 2013). Another retrospective study adding sitafloxacin (STFX) to MAI-PD patients who did not achieve culture conversion after 1 year of standard therapy reported culture conversion in 44% of patients after 6 months (Fujita et al., 2016). Quinolones such as MFLX and STFX may be effective agents for refractory MAI-PD.

*M. abscessus* requires a multidrug therapeutic approach, including injectable drugs, at treatment initiation, and maintenance treatment requires a prolonged duration. According to these guidelines, macrolide-resistant strains should receive two or more drugs during maintenance therapy. However, selection of an effective antimicrobial agent remains challenging (Daley et al., 2020). CFZ has demonstrated synergistic effects against *M. abscessus* and MAI *in vitro* when combined with AMK and CAM, making it a promising agent (van Ingen et al., 2012b; Ferro et al., 2016; Gangadharam et al., 1988). CFZ is known to cause adverse effects including QT prolongation, skin pigmentation changes, and gastrointestinal symptoms. RBT has also been reported to synergize with CAM and Tigecycline (TGC) *in vitro* (Pryjma et al., 2018). *M. abscessus* develops macrolide-induced resistance via *whiB7* and *erm* (Bich Hanh et al., 2021) gene expression, but RBT suppresses these genes *in vitro* and displays synergistic effects with macrolides (Aziz et al., 2020). This synergistic effect was not observed in the *erm41*-negative strain, suggesting that it may be an effective treatment for macrolide-resistant *M. abscessus*. In a clinical trial conducted to evaluate the efficacy of adding TGC to the initial treatment phase, the culture conversion rate was significantly higher in the TGC-treated group (89%) than in the control group (50%) after 1 month of treatment. However, no significant difference was observed between the two groups at 12 months (Kim S. R. et al., 2022). Prolonged use of TGC is complicated by its poor tolerability and the occurrence of adverse events. In an open-label prospective trial using ALIS for *M. abscessus* infection, 50% of cases achieved culture conversion within 1 year (Siegel et al., 2023). Omadacycline (OMD) has also shown synergistic effects with CAM both *in vitro* and *in vivo* (Bich Hanh et al., 2021; Fujiwara et al., 2023). In drug susceptibility studies, STFX exhibited a lower MIC than MFLX, suggesting that it may be effective (Kamada et al., 2021; He et al., 2021; Fujiwara et al., 2021). The use of a β-lactam antibiotic in combination with an appropriate β-lactamase inhibitor has been proposed as a potential treatment for *M. abscessus* infections. *M. abscessus* expresses a chromosome-encoded Ambler class A β-lactamase (Bla_Mab_), which confers resistance to various β-lactam antibiotics. The combination of avibactam and amoxicillin showed synergistic effects *in vitro* and improved survival in a zebrafish model of *M. abscessus* infection (Dubee et al., 2015). Other combinations, such as amoxicillin-imipenem-relebactam and vaborbactam in combination with carbapenems, have also demonstrated efficacy against *M. abscessus* (Lopeman et al., 2020; Kaushik et al., 2019). Although the efficacy of β-lactamase inhibitors has not yet been established in clinical trials, these findings might provide promising results for future treatment options. Given the limited number of effective agents against *M. abscessus*, continued development of new agents is anticipated.

## 3 Drug–drug interactions with comorbidities

### 3.1 Chronic pulmonary aspergillosis

The interactions between NTM-PD treatment and medication for comorbidities are shown in Table 2. RFP and RBT are potent inducers of CYP3A4, and can affect the efficacy of other therapeutic agents. Approximately 10% of NTM-PD cases are complicated by CPA, with fibrocavitary NTM-PD, COPD, and systemic steroid administration being recognized as risk factors for CPA development (Takeda et al., 2016; Phoompoung and Chayakulkeeree, 2020; Fukushima and Kida, 2021). It has been reported as an independent prognostic factor for NTM-PD (Takeda et al., 2016; Fukushima and Kida, 2021). CPA treatment in patients with NTM-PD is typically prolonged, and oral azoles are the primary therapeutic option. However, rifamycin cannot be used in combination with azoles, because it significantly reduces the concentration of azoles (Jaruratanasirikul and Sriwiriyajan, 1998; Moon et al., 2017; Geist et al., 2007; Townsend et al., 2017). The role of RFP in the treatment of NTM-PD has been questioned, and the impact of discontinuing RFP on treatment efficacy may be limited (van Ingen et al., 2024). CAM and AZM increase the blood concentration of voriconazole (VRCZ); therefore, dosage adjustment based on therapeutic drug monitoring (TDM) is required. CAM cannot be used in combination because of its significant increase in isavuconazole (ISCZ) concentration (Mushtaq et al., 2023; Purkins et al., 2003). Meta-analyses reported no difference in microbial outcomes between AZM and CAM; hence, AZM is recommended for patients receiving ISCZ (Pasipanodya et al., 2017). Given these interactions, it is recommended that rifamycin be discontinued while using azoles when NTM-PD develops with CPA, and that NTM-PD and CPA be treated simultaneously (Phoompoung and Chayakulkeeree, 2020). If rifamycin cannot be discontinued, echinocandin is an alternative, although its administration via intravenous infusion makes its long-term use difficult. Currently, global clinical trials are being conducted on Fosmanogepix and Olorofim, which can be used as alternatives to azoles. In a phase one study examining the interaction between Fosmanogepix and RFP, the addition of RFP resulted in a slight decrease in the AUC of Fosmanogepix, although the effect was limited and could be managed with dose adjustment (Hodges et al., 2024). Olorofim is a weak inhibitor of CYP3A4 that may interact with rifamycin, although the degree of interaction is lower than that observed with azoles (Rauseo et al., 2020).

**Table 2:** Drug–drug interactions between non-tuberculous mycobacterial infections and coexisting diseases.

	Concomitant drugs	Antimicrobial agents	Interactions	References
Azoles	ITCZ	RFP	RFP decreases AUC of ITCZ 88%.	Jaruratanasirikul and Sriwiriyajan (1998)
		RBT	RBT decreases the serum concentration of ITCZ 81%.	Moon et al. (2017)
	VRCZ	RFP	RFP decreases Cmax of VRCZ 93%.	Geist et al. (2007)
		CAM	CAM increases Cmax of VRCZ 52%.	Mushtaq et al. (2023)
		AZM	AZM increases Cmax of VRCZ 17%.	Purkins et al. (2003)
	ISCZ	CAM	CAM (strong CYP3A/5 inhibitor) may increase ISCZ concentration.	not studied
		RFP	RFP decreases Cmax of ISCZ 75%.	Townsend et al. (2017)
Immunosuppressants	Prednisolone	RFP	RFP decreases AUC of Prednisolone 66%.	McAllister et al. (1983)
	Tacrolimus	RFP	RFP increases the clearance of tacrolimus 47%.	Hebert et al. (1999)
		RBT	RBT decreases the serum concentration of tacrolimus 47%.	Kim O. H. et al. (2022)
		CAM	CAM increases AUC of tacrolimus by 4-fold.	Wen et al. (2023)
Anticoagulants	Warfarin	RFP	Monitor prothrombin time; may require 2- to 3-fold warfarin dose increase.	Nahid et al. (2016)
Cardiovascular agents	Calcium blocker	RFP	Clinical monitoring recommended; may require change to an alternate cardiovascular agent.	Nahid et al. (2016)
	Digoxin	RFP	TDM recommended; may require digoxin dose increase.	Nahid et al. (2016)
Theophylline	Theophylline	RFP	TDM recommended; may require theophylline dose increase.	Nahid et al. (2016)
Hypolipidemics	Statins	RFP	Monitor hypolipidemic effect; may require use of an alternate antihyperlipidemic drug.	Nahid et al. (2016)
PI	ATV(unboosted)	RBT	ATV may increase RBT AUC.	Department of Health and Human Services, 2024 Guidelines
	ATV/r	RBT	ATV/r increases RBT AUC 110% and metabolite AUC 2101%.	
	DRV/r	RBT	DRV/r does not change RBT AUC and increases metabolite AUC 881%.	
	LPV/r	RBT	LPV/r increases RBT AUC 203% and metabolite AUC 375%.	
	PI/c	RBT	PI/c may increase RBT. RBT may decrease COBI.	
	All PIs	RFP	RFP decreases PI concentration by >75%.	
NNRTI	DOR	RBT	RBT decreases DOR AUC 50%.	
		RFP	RFP decreases DOR AUC 88%.	
	EFV	RBT	EFV decreases RBT concentration 38%.	
		RFP	RFP decreases EFV AUC 26%.	
	ETR	RBT	ETR does not change RBT and metabolite AUC. RBT decreases ETR AUC 37%.	
		RFP	RFP decreases ETR concentration.	
	NVP	RBT	NVP increases RBT AUC 17% and metabolite AUC 24%. RBT decreases NVP Cmin 16%.	
		RFP	RFP decreases NVP concentration 20–58%.	
	RPV PO	RBT	RBT does not change RPV AUC and Cmin.	
		RFP	RFP decreases RPV AUC 80%.	
NRTI	TAF	RBT	RBT may decrease TAF concentration.	
		RFP	RFP decreases TAF AUC 55%.	
	TDF	RBT	RBT does not change TFV AUC.	
		RFP	RFP does not change TFV AUC.	
INSTI	BIC	RBT	RBT decreases BIC AUC 38% and Cmin 56%.	
		RFP	RFP decreases BIC AUC 75%.	
	CAB PO	RBT	RBT decreases CAB AUC 23% and Cmin 26%. CAB does not change RBT concentration.	
		RFP	RFP decreases CAB AUC 59% and Cmin 50%.	
	DTG	RBT	RBT does not change DTG AUC and decreases Cmin 30%.	
		RFP	RFP increases DTG AUC 33% and Cmin 22%.	
	EVG/c	RBT	RBT decreases EVG AUC 21% and Cmin 67%. EVG/c does not change RBT AUC.	
		RFP	RFP may decrease EVG and COBI concentrations.	
	RAL	RBT	RBT increases RAL AUC 19% and decreases Cmin 20%.	
		RFP	RFP decreases RAL AUC 40% and Cmin 61%.	
CCR5 antagonist	MVC	RBT	RBT does not change MVC AUC and decreases Cmin 30%.	
		RFP	RFP decreases MVC AUC 63%.	
HIV-gp120-directed attachment inhibitor	FTR	RBT	RBT increases TMR AUC 66%.	
		RFP	RFP may decrease TMR concentration.	
Capsid inhibitor	LEN	RBT	RBT may decrease LEN concentration.	
		RFP	RFP decreases LEN AUC 84%.	

ATV, atazanavir; AUC, area under the curve; AZM, azithromycin; BIC, bictegravir; CAB, cabotegravir; CAM, clarithromycin; Cmax, maximum plasma concentration; Cmin, minimum plasma concentration; COBI, cobicistat; DOR, doravirine; DRV/r, darunavir/ritonavir; DTG, dolutegravir; EFV, efavirenz; ETR, etravirine; EVG/c, elvitegravir/cobicistat; FTR, fostemsavir; INSTI, integrase strand transfer inhibitor; ISCZ, isavuconazole; ITCZ, itraconazole; LEN, lenacapavir; LPV/r, lopinavir/ritonavir; MVC, maraviroc; NNRTI, non-nucleoside reverse transcriptase inhibitor; NRTI, nucleoside reverse transcriptase inhibitor; NVP, nevirapine; PI, protease inhibitor; PI/c, protease inhibitor/cobicistat; RAL, raltegravir; RBT, rifabutin; RFP, rifampicin; RPV, rilpivirine; TAF, tenofovir alafenamide; TDF, tenofovirdisoproxil fumarate; TMR, temsavir; VRCZ, voriconazole.

### 3.2 Immunosuppressants

An elevated incidence of NTM and increased mortality rates have been reported in patients with rheumatoid arthritis and those using immunosuppressants (Brode et al., 2015; Marras et al., 2018; Uno et al., 2020; Yeh et al., 2014). Additionally, increased mortality due to NTM-PD caused by rapidly growing mycobacteria has been documented in patients receiving anti-TNF-α inhibitors (Winthrop et al., 2009; Mori and Sugimoto, 2012). It is crucial for patients using these immunosuppressive agents to be aware of drug–drug interactions, as they are being treated for NTM-PD concurrently with their underlying diseases. RFP lowers the AUC of prednisolone by 66%, necessitating an increase in prednisolone dose (McAllister et al., 1983). Rifamycin decreased the blood concentration of tacrolimus, whereas CAM enhanced its AUC by approximately 4-fold. Therefore, TDM should be performed and adjusted to maintain appropriate blood levels (Hebert et al., 1999; Kim O. H. et al., 2022; Wen et al., 2023).

### 3.3 AIDS

Mycobacterial infections are prevalent in patients with AIDS. In a large cohort study of HIV-positive individuals in North America, disseminated MAI infection was reported to be the third most prevalent opportunistic infection following *Pneumocystis jirovecii* pneumonia and esophageal candida (Buchacz et al., 2016). Disseminated MAI infection tends to develop within 90 days of initiating antiretroviral therapy (ART; Yen et al., 2019). Some AIDS patients may require treatment for NTM disease while on ART, and rifamycin may interact with antiviral drugs because of its strong CYP3A4 induction. Although RFP is not recommended for certain combinations, RBT can be used in certain cases. Thus, the reference of the Department of Health and Human Services, 2024 guidelines is recommended.

### 3.4 Lifestyle-related diseases

RFP is a potent inducer of CYP3A4 that can interact with various drugs. A significant proportion of patients with NTM-PD are elderly and may have various lifestyle comorbidities. The tuberculosis guidelines provide information on the potential interactions between RFP and lifestyle medications (Nahid et al., 2016). Warfarin, for example, necessitates a dose increase due to its diminished concentration, while calcium blockers exhibit reduced efficacy. Similarly, the diminished efficacy of digoxin necessitates TDM to adjust its dosage. Theophylline also demonstrates reduced efficacy, which necessitates TDM to adjust its dosage. The reduced blood levels of statins resulting from RFP necessitate blood tests to evaluate their effectiveness. If statins appear ineffective, switching to another antihyperlipidemic drug may be required.

## 4 Discussion

Macrolides are crucial medications for the treatment of NTM-PD, and EB is an essential drug for suppressing macrolide resistance. To prevent interruptions in EB treatment due to adverse events, adjustments such as intermittent dosing in non-cavitary NB-type cases and low-dose EB administration should be considered (Jeong et al., 2015; Ando et al., 2021). The impact of RFP in non-HIV MAI-PD patients may be limited, and discontinuation may be considered when concomitant use is difficult (van Ingen et al., 2024). The significance of RFP administration awaits the outcomes of the ongoing RCT. In contrast, CFZ regimens may be effective; however, RCT results are limited. ALIS has demonstrated efficacy in refractory MAI-PD, but AMK has been reported to be resistant in 10% of cases after ALIS administration (Griffith et al., 2018). Additionally, the knowledge regarding which patients are likely to respond to treatment and the appropriate treatment duration is limited. In refractory MAI-PD, the addition of BDQ, CFZ, or STFX may be considered as an alternative treatment option in cases where ALIS is less effective. Effective agents for the maintenance treatment of *M. abscessus* are currently limited, and there is a need for new agents such as OMD and STFX.

Higher complication rates have been reported for CPA in patients with cavities as well as in those with COPD and immunosuppressive drug use (Phoompoung and Chayakulkeeree, 2020). Given the poor prognosis associated with CPA, prioritizing treatment is crucial (Takeda et al., 2016; Phoompoung and Chayakulkeeree, 2020; Fukushima and Kida, 2021). In some cases of CPA development, RFP must be discontinued because of its interaction with azoles. Fosmanogepix and Olorofim, which are currently in clinical trials and exhibit reduced susceptibility to CYP3A4 compared with azoles, are promising CPA treatments that have developed into NTM-PD (Hodges et al., 2024; Rauseo et al., 2020). For patients receiving immunosuppressants, TDM should be performed to adjust the optimal dose or RFP should be discontinued. Additionally, reviewing the latest HIV guidelines can help determine the appropriate concomitant medications. Considering the long-term nature of NTM disease treatment, which often requires multidrug therapy, it is essential to understand the characteristics of each drug and be aware of the potential drug interactions.

This mini-review has several limitations. First, there is a lack of information regarding drug–drug interactions related to newly developed drugs under clinical trials; therefore, it has not been described. Second, there is no discussion on the interactions of NTM-PD drugs with lifestyle-related diseases such as hypertension and dyslipidemia. Third, owing to the extensive number of medications used in ART for HIV, we limited the description to the table.

## 5 Conclusion

This mini-review provided an overview of the synergistic effects associated with conventional therapies for NTM-PD, and their interactions with medications used to treat other conditions. Since many NTM-PD patients are elderly, the frequency of treatment-related adverse events is high, and interactions with medications to treat complications can be problematic. It is essential to carefully evaluate drug–drug interactions and the potential for adverse events when selecting appropriate treatment regimens.
